# NEIGHBORHOOD SOCIOECONOMIC STATUS AND SARCOPENIA IN COMMUNITY-DWELLING OLDER ADULTS

**DOI:** 10.1093/geroni/igae098.4030

**Published:** 2024-12-31

**Authors:** Xiangyu Zhai, Moyao Wang, Shiyin Zhan, Doris Sau Fung Yu

**Affiliations:** The University of Hong Kong, Hong Kong, China (People’s Republic); The University of Hong Kong, Hong Kong, China (People’s Republic); The University of Hong Kong, Hong Kong, China (People’s Republic); The University of Hong Kong, Hong Kong, China (People’s Republic)

## Abstract

Individual-level socioeconomic characteristics are known to predict sarcopenia, but less is known about how the neighborhood context shapes this debilitating problem among older adults. This study aims to examine the association between nSES and sarcopenia. We included 943 older adults aged over 60 years (56.0% female, mean age 72 ± 8.1). Sarcopenia was diagnosed according to the Asian Working Group for Sarcopenia 2019 (AWGS2019) algorithm covering skeletal muscle mass, muscle strength, and physical performance. nSES, the sum of the standardized values of the proportion of single-person households, never-married status, low education attainment, households with low income, unemployment, non-professional vocation, and non-owner occupiers, were obtained from the Census and Statistics Department and divided into distribution-based tertiles of all 292 Tertiary Planning Units in Hong Kong (T1 = highest nSES). There were 89 participants (8.4%) diagnosing sarcopenia. The sample was distributed across three tertiles of nSES (T1: 157 participants [16.6%]; T2: 193 participants [20.5%]; T3: 593 participants [62.9%]). Compared with T1, the lower nSES tertiles showed higher odds of sarcopenia, after adjusting for age, sex, body mass index, and individual-level socioeconomic characteristics (T2: OR = 1.38 [95% CI, 0.49 – 3.87], T3: OR = 3.53 [95% CI, 1.51 – 8.28], T2: aOR = 1.53 [0.51 – 4.54], T3: aOR = 3.12 [95% CI, 1.21 – 8.03]). This is the first study to identify the negative relationship between comprehensive nSES and the prevalence of sarcopenia. The findings urge the need to develop socio-environmental sensitive risk stratification and preventive care to manage later-age sarcopenia.

